# Forearm lengthening: management of elbow and wrist

**DOI:** 10.1007/s11832-016-0786-9

**Published:** 2016-11-08

**Authors:** Franck Launay, Sébastien Pesenti

**Affiliations:** Service des Urgences Pédiatriques, Hôpital Timone Enfants, 264 rue St-Pierre, 13005 Marseille, France

**Keywords:** Forearm, Lengthening, Radial club hand, Multiple exostoses disease

## Abstract

The risk and consequences of an elbow or a wrist contracture are lower during a forearm lengthening than during a lower limb lengthening. This kind of complication can mostly be avoided by an active and intensive regimen of physiotherapy. However, there are some challenges to deal with in treating the disorder multiple exostoses and the radial club hand, including the lack of consensus on the best treatment for multiple exostoses. However, it is important to realize that the evolution of multiple exostoses can lead to a radial head dislocation which will damage the pronation and the supination range of motion. As this motion can be poor even without a radial head dislocation as a result of the radius being longer than the ulna, an interesting technique can be to lengthen the ulna to limit this phenomenon. In radial club hand, the main problem is the deviation of the hand requiring a centralization. The best treatment for centralization of the hand is to do a progressive correction with an external fixator. Thereafter, it is possible to lengthen the forearm, but this indication is mainly cosmetic in the unilateral radial club hand.

The indications for an upper limb lengthening are less common than those for a lower limb lengthening. Consequently, the management, indications, risks, and potential complications of procedures for lengthening of the upper limb, as well as the results of these procedures, are less well codified than those for a femoral and/or tibial lengthening.

There are so many different conditions which can require a lengthening of the forearm, and the problems in the elbow and wrist which can result from these conditions are specific for each condition.

One of the major differences between forearm and lower limb lengthening is the subsequent need for physiotherapy, primarily because there are fewer joint contractures during forearm lengthening than lower limb lengthening. In the elbow, flexion depends on the action of four muscles, namely, the biceps, brachialis, brachioradialis, and pronator teres, with the most important and the strongest of these being the biceps and the brachialis muscles. Therefore, in a forearm lengthening procedure, the osteotomy is necessarily below the distal insertion of these both latter two muscles. As it is evident that elbow flexion contracture cannot occur in this condition, it is not necessary to perform any tenotomy around the elbow before or during the upper limb lengthening surgery.

Conversely, the management of the wrist has to be more active because there is a real risk of flexion contracture of the wrist and of the fingers. However, the risk is less important than in the lower limb. Thus, simple physiotherapy and a part-time brace are most often sufficient measures to prevent or to treat a flexion contracture of the wrist.

The more frequent indications of forearm lengthening in children relate to the genetic condition of multiple exostoses and to radial club hand. Individuals with multiple exostoses have a shortening of the ulna in comparison to the radius, with the development of an exostosis from the distal ulna. This condition leads to a decreased pronation and supination range of motion and to an ulna deviation of the wrist. The affected individual will subsequently develop radial bowing and ultimately dislocation of the radial head, which in turn will worsen the pronation and supination range of motion still further, as well as elbow flexion.

A search of the literature reveals that there is no consensus on the best treatment for multiple exostoses, with recommended treatments ranging from the simple resection of an exostosis of the distal ulna to a complex lengthening of the ulna associated with a distal radius osteotomy, an ulnar exostosis resection, and an annular ligament reconstruction. This profusion of published treatments indicates a serious lack of informed knowledge on the best manner to treat this condition. Consequently, the treating physician has to be honest with the patient and the family with respect to the attainable results of the surgery [1]. We believe that the resection of the distal ulnar exostosis will likely improve the pronation and the supination and that the lengthening of the ulna will most certainly improve the ulnar deviation of the wrist. However, the result of such surgery on the pronation and supination motion is not truly predictable.

One major goal of ulna lengthening is to avoid the progressive bowing of the radius and the dislocation of the radial head. Thus, lengthening of the ulna in the case of dislocation of the radial head will improve the flexion extension range of motion, but once again, how the result will affect the pronation and the supination range of motion is not predictable.

Finally, when the radial head is dislocated in this condition, it is unrealistic to believe that a simple osteotomy of the ulna will be sufficient to reduce the radial head. As the ulna is too short, the best strategy to manage the dislocation is to lengthen the ulna [2] in order to move the radial head away from the lateral condyle, thus improving the pronoation–supination range of motion.

In the case of the radial club hand, the main problem is the extreme radial deviation of both the hand and wrist (Fig. 1). In the newborn, the first treatment is based on braces and physiotherapy to maintain the reduction of the wrist, if the reduction is possible, or to improve the reduction if it is impossible to reduce it. With surgery, the first step is pollicization of a finger in order to reconstruct a thumb. Then comes the timing of the centralization. With no deviation of the hand, which is a very rare condition, there is nothing to do except maintain a close follow-up during growth. However, if there is a radial deviation, and if this deviation is reducible, the centralization can be done with a pin bridging the wrist. This pin can extend from the olecranon to one of the metacarpal bones and must be replaced during growth because it will become too short and too thin, leading to breakage. One trick to know about the pinning: it can be interesting to put a screw in the olecranon in order to fix the proximal part of the pin (Fig. 2), as this will prevent the pin from sliding completely in the olecranon during growth. It would seem the better option that the pin slides into the metacarpal bone because it will be easier to remove it the pin needs replacing.

In many cases the wrist will subsequently progress to an arthrodesis, but the pin allows the growth of the wrist during childhood.

**Fig. 1:**
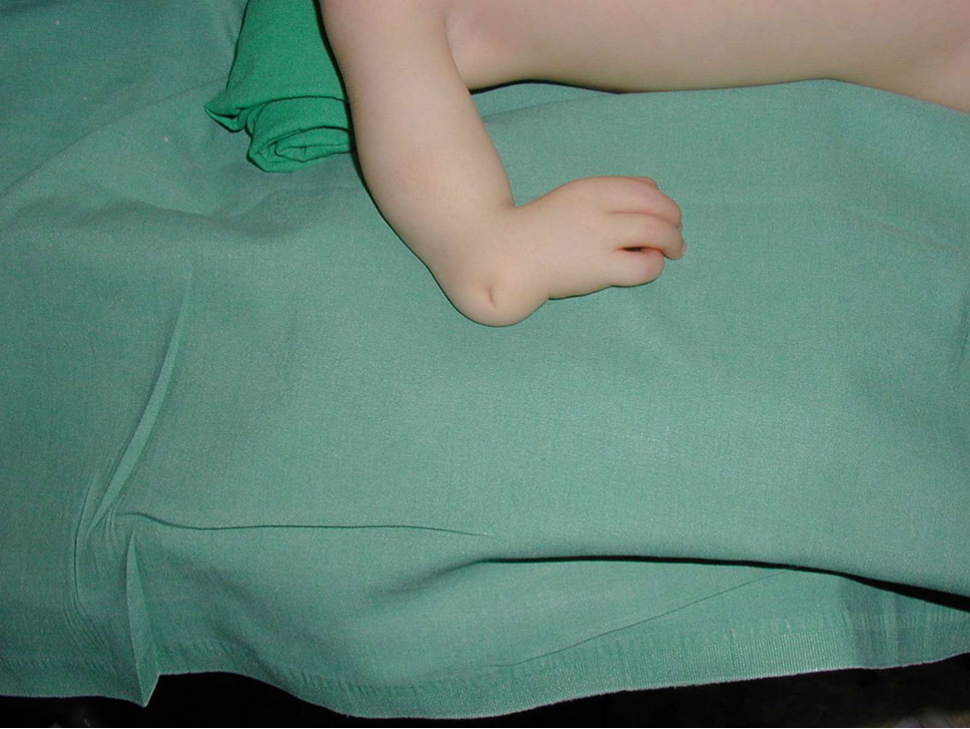
Typical hand deviation in a radial club hand

**Fig. 2:**
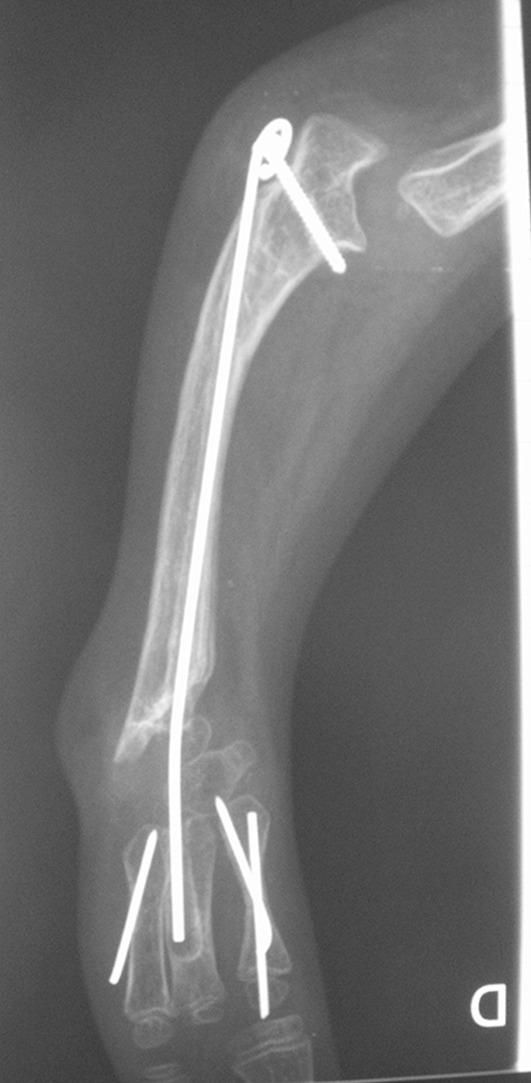
Pin bridging the wrist in a radial club hand with a screw in the olecranon

On the other hand, if the hand is not reducible, there are two possibilities to centralize the hand. The first one is to reduce the wrist by releasing surgically the soft tissues around the wrist and to bring the carpal bones in front of the ulnar head, which then allows the pinning. The second approach is to reduce the wrist progressively with a fixator without any soft tissue release, possibly with a monolateral frame [3] or an Ilizarov frame (Fig. 3), but it is a very good indication for a hexapod frame. The hexapod frame will allow not only correction of the radial deviation but also correction of the anterior dislocation of the carpal bones. When the reduction is achieved, one can place the pin bridging the wrist and then remove the frame.

**Fig. 3:**
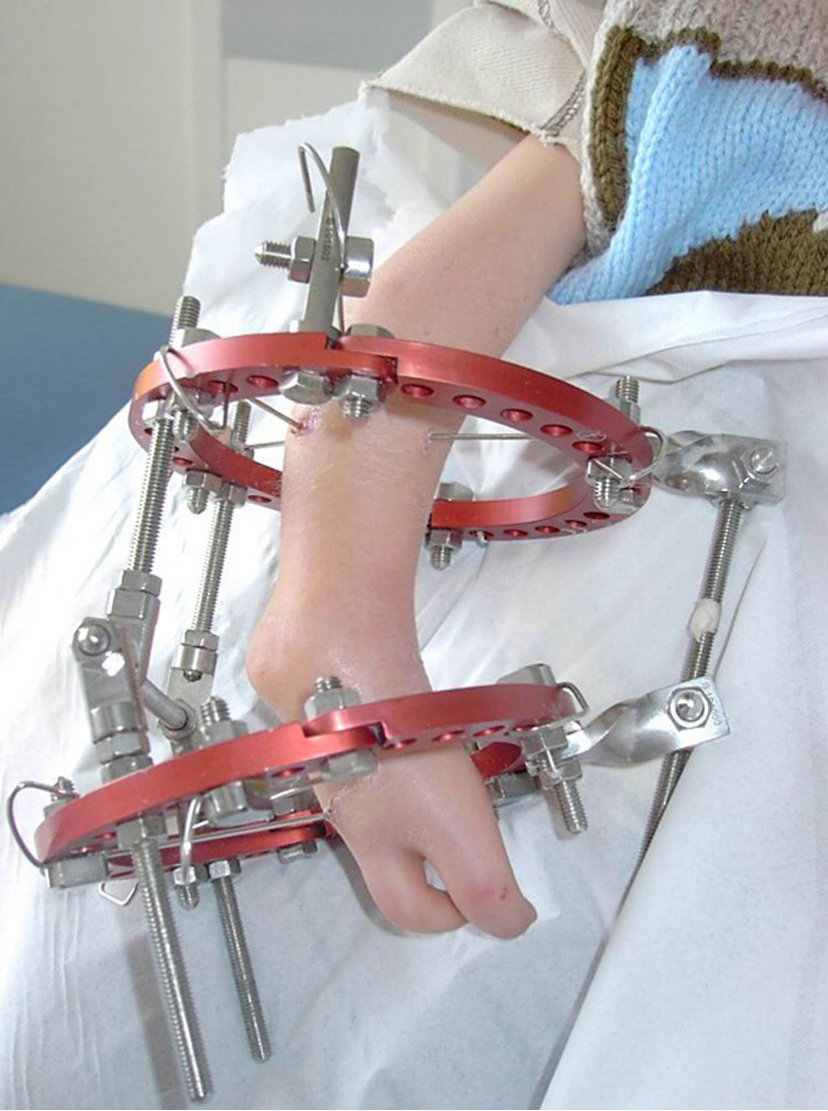
Progressive centralization of the hand in a radial club hand using an Ilizarov frame

Later on during childhood, there is often a demand for a forearm lengthening, which will be a cosmetic indication in the case of an unilateral radial club hand and a functional indication in the case of a bilateral radial club hand. The elbow presents no major problem during lengthening, but the wrist has to be protected with a pin through the wrist if the wrist is not already arthrodesed, or by extending the frame to the hand. Intensive physiotherapy has to be followed during the procedure to avoid flexion contracture of the fingers associated with bracing. Finally, even if the initial indication for lengthening is cosmetic, after this procedure the patient will experience an improvement of muscle strength with a better grip.

In conclusion, the wrist represents the major challenge during a forearm lengthening. The goal of the treatment is not to provide a good motion of this joint but to have the hand correctly aligned with the forearm to allow the patient to have the correct function.
